# Immune inactivation of anti-simian immunodeficiency virus chimeric antigen receptor T cells in rhesus macaques

**DOI:** 10.1016/j.omtm.2021.06.008

**Published:** 2021-06-24

**Authors:** Françoise Haeseleer, Yoshinori Fukazawa, Haesun Park, Benjamin Varco-Merth, Blake J. Rust, Jeremy V. Smedley, Karsten Eichholz, Christopher W. Peterson, Rosemarie Mason, Hans-Peter Kiem, Mario Roederer, Louis J. Picker, Afam A. Okoye, Lawrence Corey

**Affiliations:** 1 Department of Laboratory Medicine, University of Washington, Seattle, WA, USA; 2 Vaccine and Infectious Disease Division, Fred Hutchinson Cancer Research Center, Seattle, WA, USA; 3 Vaccine and Gene Therapy Institute and Oregon National Primate Research Center, Oregon Health & Science University, Beaverton, OR, USA; 4 Stem Cell and Gene Therapy Program, Fred Hutchinson Cancer Research Center, Seattle, WA, USA; 5 Department of Medicine, University of Washington, Seattle, WA, USA; 6 Vaccine Research Center, National Institute of Allergy and Infectious Diseases, National Institutes of Health, Bethesda, MD, USA

**Keywords:** CAR T cells, HIV, SIV, infectious diseases, humoral immune response, autologous cell therapy

## Abstract

Chimeric antigen receptor (CAR) T cell therapies are being investigated as potential HIV cures and designed to target HIV reservoirs. Monoclonal antibodies (mAbs) targeting the simian immunodeficiency virus (SIV) envelope allowed us to investigate the potency of single-chain variable fragment (scFv)-based anti-SIV CAR T cells. *In vitro*, CAR T cells expressing the scFv to both the variable loop 1 (V1) or V3 of the SIV envelope were highly potent at eliminating SIV-infected T cells. However, in preclinical studies, *in vivo* infusion of these CAR T cells in rhesus macaques (RMs) resulted in lack of expansion and no detectable *in vivo* antiviral activity. Injection of envelope-expressing antigen-presenting cells (APCs) 1 week post-CAR T cell infusion also failed to stimulate CAR T cell expansion *in vivo*. To investigate this *in vitro* versus *in vivo* discrepancy, we examined host immune responses directed at CAR T cells. A humoral immune response against the CAR scFv was detected post-infusion of the anti-SIV CAR T cells; anti-SIV IgG antibodies present in plasma of SIV-infected animals were associated with inhibited CAR T cell effector functions. These data indicate that lack of *in vivo* expansion and efficacy of CAR T cells might be due to antibodies blocking the interaction between the CAR scFv and its epitope.

## Graphical abstract

**Figure:**
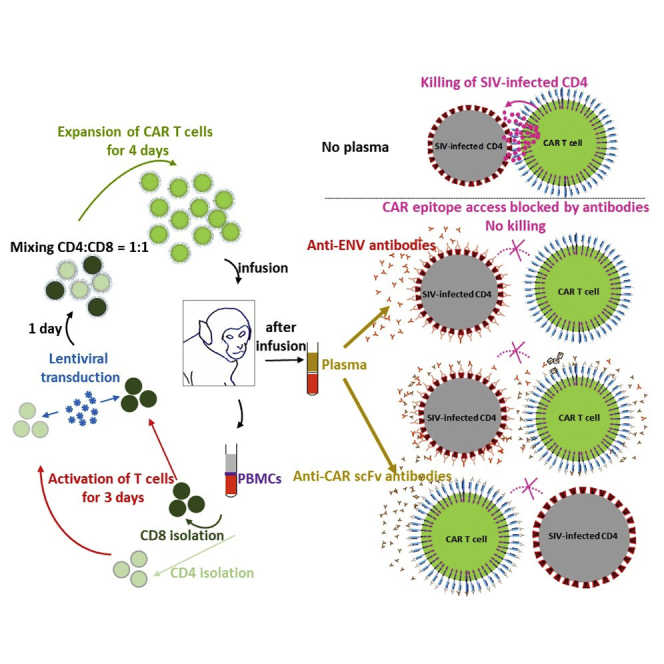

## Teaser

Corey and colleagues evaluated CAR T cells targeting the envelope of simian immunodeficiency virus. They show that antibodies blocking the interaction between the CAR and its epitope might be responsible for the lack of *in vivo* proliferation of anti-SIV CAR T cells and killing of SIV-infected cells after infusion in rhesus macaques.

## Introduction

The technologies to characterize and manufacture broadly neutralizing antibodies (bnAbs) to HIV have broadened the potential for using immunological approaches to treat and prevent HIV infection. Clinical development of bnAbs for both their antiviral function and prevention of HIV is underway, and several successes have been seen with such antibodies. Access to the DNA sequence analyses of these bnAbs has paved the way for their use in developing chimeric antigen receptor (CAR) T cells to simian immunodeficiency virus (SIV)/simian-human immunodeficiency hybrid virus (SHIV)/HIV.^1^^,^^2^

Several clinic trials with autologous T cells from patients that had been engineered to express a CAR have demonstrated the potential of CAR T cell therapy against cancer, especially for hematologic malignancies.^3^, ^4^, ^5^ Adoptive cell therapy has also been investigated as a strategy to eliminate HIV-infected cells.^6^^,^^7^ The therapeutic potential of such an approach has been primarily studied with CD4CAR constructed with the extracellular domain of the human CD4 receptor that binds to the HIV envelope (Env).^8^, ^9^, ^10^, ^11^ Other HIV-specific CARs were engineered with the single-chain variable fragment (scFv) of bnAbs recognizing a conserved epitope of the HIV Env.^12^ Although some success was obtained with CD4CAR in controlling viremia to lower levels, eradication of the HIV reservoirs using CAR T cells has not yet been accomplished.^13^^,^^14^

We developed a proof-of-concept preclinical program to evaluate if CAR T cells constructed with the scFv of rhesus macaque (RM) bnAbs could exert *in vivo* immunological control of SIV-infected T cells. After selection of potent *in vitro* scFv-based CAR T cells, we developed a vector that can provide protection to the CAR T cells from SIV infection and promote cell trafficking to the B cell follicles.^15^ After infusion of the CAR T cells in RMs, we did not observe expansion of the CAR T cells. We did, however, identify an antibody-mediated immune response against the CAR in one of the infused RMs that might have contributed to the failure of the CAR T cells to survive and proliferate.

## Results

### Construction and optimization of CAR lentiviral vector

**Figure 1:**
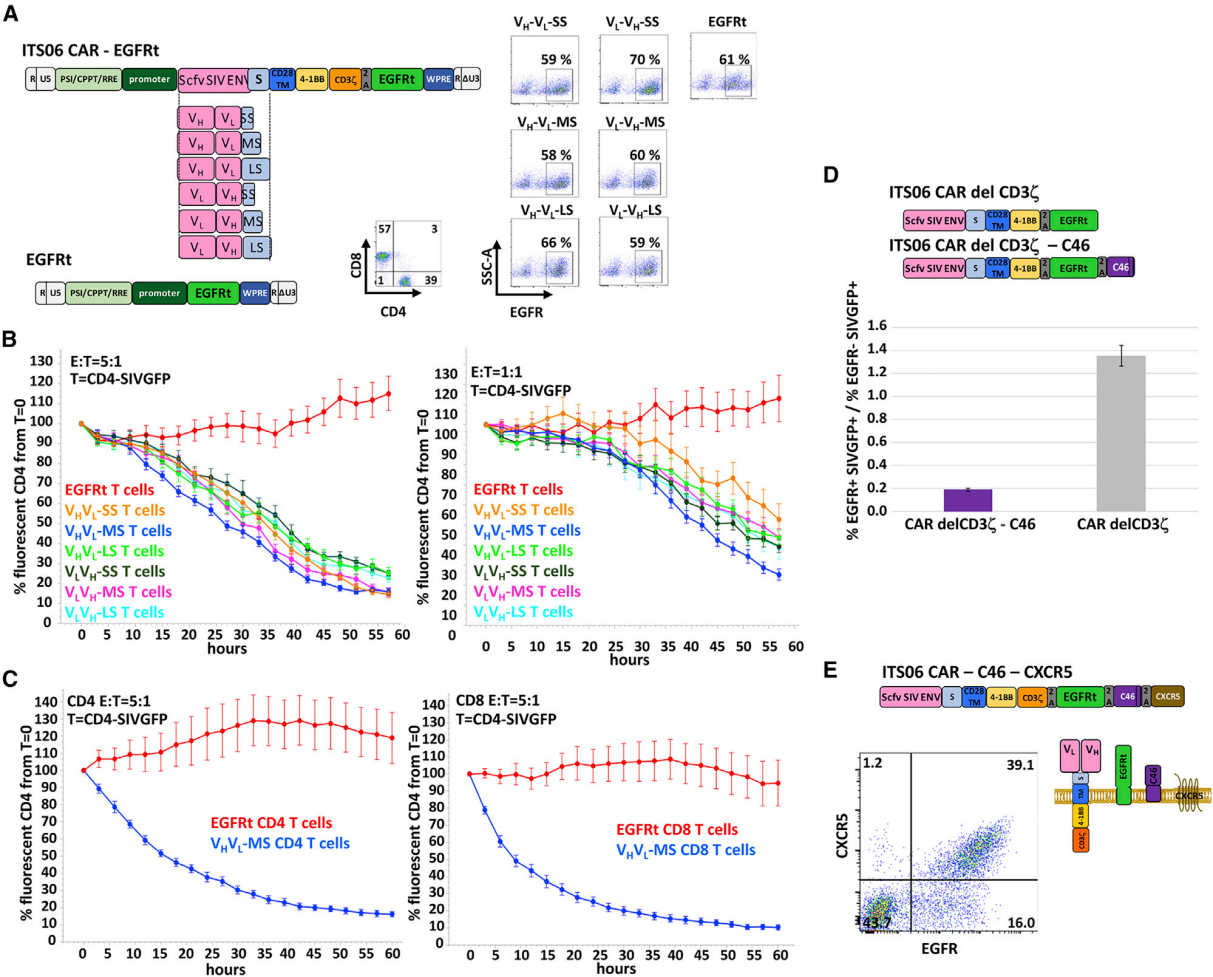
Optimization of the CAR lentiviral vector (A) Schematic diagram of the anti-SIV Env CAR with the scFv of the anti-Env ITS06 antibody in the V_H_-V_L_ or V_L_-V_H_ orientation linked through a short (SS), medium (MS), or long (LS) spacer to the CD28 transmembrane domain (left). Flow cytometry analysis of CAR T cells transduced with the SIV Env CAR-EGFR or EGFR lentiviral vectors is shown (right). Numbers in the dot plots indicate the percentage of gated cells. (B) Real-time detection of fluorescent SIV-infected CD4^+^ targets in the presence of CAR T cells or control EGFR T cells shown in (A). Images of triplicate wells were taken every 3 h and analyzed with the IncuCyte image analysis software. The percentage of SIVGFP-infected CD4^+^ T cells relative to their number at time T = 0 after addition of anti-SIV CAR T cells or EGFR T cells is indicated over time at the E:T ratios of 5:1 or 1:1. The error bars indicate the standard error to the mean. (C) Killing of SIVGFP-infected CD4^+^ targets in the presence of isolated CD4^+^ or CD8^+^ T cells transduced with the ITS06-V_H_-V_L_-MS CAR were analyzed as in (B). (D) Analysis of protection of CD4^+^ T cells from SIV infection. Schematic diagram of the lentivirus vector encoding the ITS06-V_H_-V_L_-MS CAR deleted of its CD3ζ signaling domain with or without the C46 fusion inhibitor. CD4^+^ T cells transduced with these ITS06 CAR variants were incubated with SIVGFP viruses. The percentage of fluorescent infected cells was determined by flow cytometry and compared in EGFR+ and EGFR− CD4^+^ T cells. (E) Schematic diagram of the fully optimized lentiviral vector encoding all four ITS06 CAR, EGFR, C46, and CXCR5 proteins and of their expression at the cell surface. Flow cytometry analysis of CD3 T cells transduced with the optimized lentivirus for the coexpression of EGFR and CXCR5.

Similar to humans and HIV, predominant targets of anti-SIV bnAbs are the variable loop 2 (V2) and V3 regions of the Env. We obtained the sequence of high-affinity anti-SIV antibodies, ITS01, ITS06.01, or ITS52, targeting the CD4 binding site (ITS01), V1 (ITS06.01), or V3 (ITS52) of SIV Env^2^ and cloned each scFv. As a first step in designing anti-SIV CAR lentiviral vectors, we investigated whether the configuration of the V_H_ (variable domain of the heavy chain) and V_L_ (variable domain of the light chain) domains of the scFv or the length of the spacers linking the scFv to the transmembrane domain have an effect on the overall potency of the CAR T cells. Using the scFv of the ITS06.01 antibody, we constructed three lentiviral vectors with the V_H_ and V_L_ configuration or the V_L_ and V_H_ configuration and spacers of 12, 119, or 228 amino acids consisting of the human IgG4 hinge, hinge-CH3, or hinge-CH2-CH3 domains, respectively (Figure 1A).^16^^,^^17^ These extracellular domains were linked to a CD28 transmembrane domain, a 4-1BB intracellular costimulatory domain, and a CD3ζ activation domain. To assess the efficiency of lentiviral transduction into T cells, the DNA constructs also included a truncated version of the epidermal growth factor receptor (EGFR) that can be detected on the cell surface using an anti-EGFR monoclonal antibody (mAb) (cetuximab). Purified CD4^+^ and CD8^+^ T cells mixed at a ratio of about 1:1 were transduced with these lentiviral vectors and expressed similar amounts of EGFR as determined by flow cytometry (Figure 1A).

The killing potency of anti-SIV CAR T cells was evaluated using an IncuCyte cytotoxic assay that records in real time the disappearance of CD4^+^ target cells infected with a SIVmac239 virus carrying an enhanced green fluorescent protein (EGFP) gene (SIVGFP).^18^ Although CAR T cells with a V_H_-V_L_ orientation combined with a medium spacer were slightly more potent, all combinations of spacers and scFv domains generated CAR T cells with similar killing potency toward SIV-infected targets (Figure 1B). No significant killing of control EGFR T cells was observed. In addition to producing cytokines and showing synergistic antitumoral activity with CD8^+^ T cells, CD4^+^ T cells have also been shown to have comparable cytotoxic activities to CD8^+^ T cells.^19^^,^^20^ Our CAR T cell products are composed of a mix of CD4^+^ and CD8^+^ cells at a ratio of about 1:1, with both CD4^+^ and CD8^+^ CAR T cells capable of killing SIV-infected cells with similar potency (Figure 1C).

Because CD4^+^ CAR T cells are susceptible to SIV infection, they might not only contribute to the viral load but could also become targets of CAR T fratricide. We introduced a membrane-anchored fusion inhibitory peptide derived from the gp41 (C46) to protect the CD4^+^ CAR T cells against SIV infection.^21^ To discriminate between a reduced number of SIV-infected cells due to CAR T cell-mediated killing or due to protection provided by C46, we tested the protective efficacy of C46 with an ITS06 CAR deleted of the CD3ζ domain (Figure 1D). CD4^+^ T cells were transduced with the ITS06-delta CD3ζ with or without the C46 and infected with SIV-GFP 7 days later. SIV infection was detected by flow cytometry for GFP expression 3 days post-infection and protection assessed by comparing the percentage of GFP+ cells among transduced EGFR+ cells and untransduced EGFR− cells. In the absence of protection by the C46 peptide (ITS06-deltaCD3ζ), the ratio of the percentage of GFP+ cells among EGFR+ cells over the percentage of GFP+ cells among EGFR− cells was around 1, because EGFR+ and EGFR− cells would be similarly susceptible to infection (Figure 1D). In contrast we observed up to a 7-fold decrease in SIV infection when the cells were transduced with ITS06-deltaCD3ζ-C46 (Figure 1D).

For CAR T cell trafficking to B cell follicles where target cells that are major sites of SIV/HIV persistence reside,^22^, ^23^, ^24^ we added the cDNA encoding rhesus CXCR5, a homing receptor shown to promote cell trafficking to B cell follicles in lymph nodes of RMs, to our lentiviral vector.^15^^,^^25^ About 39% out of 55.1% EGFR+ transduced T cells expressed both EGFR and CXCR5 (Figure 1E). These data demonstrate that with this single lentiviral vector we can generate potent anti-SIV Env CAR T cells that express the homing receptor CXCR5 and are protected against SIV infection.

### Comparison of killing potency and proliferation of ITS01-, ITS06-, and ITS52-based CAR T cells *in vitro*

**Figure 2:**
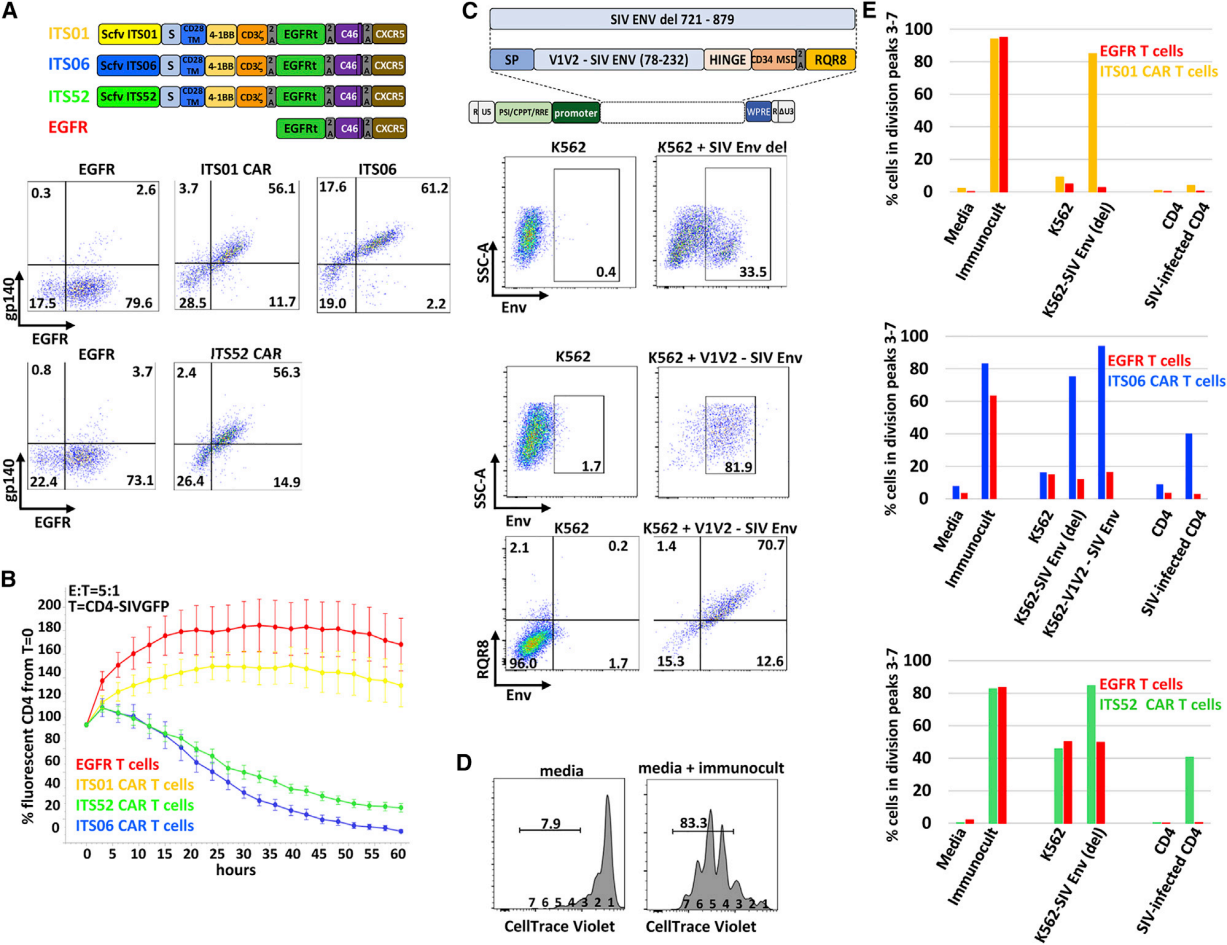
Comparison of killing potency and proliferation of ITS01, ITS06, and ITS52-based CAR T cells *in vitro* (A) Anti-SIV CARs based on the ITS01, ITS06, and ITS52 anti-SIV Env monoclonal antibodies bind the SIVmac239 gp140. Schematic diagram of the anti-SIV Env CAR. CD8^+^ T cells transduced with the anti-SIV CAR were incubated with SIVmac239 gp140 proteins followed by staining with biotinylated anti-Env mAbs and BV421-streptavidin. (B) Comparison of the killing potency of ITS01, ITS06, and ITS52 CAR T cells toward SIVGFP-infected CD4 T cells using a real-time killing assay as described in Figure 1B. The error bars indicate the standard error to the mean. (C) Characterization of Env-expressing APCs. Schematic diagram of the lentiviral vectors encoding the full extracellular domain or the V1V2 region of the SIVmac239 EnvV. The lentiviral vector encoding the V1V2 Env also included the RQR8 marker. Flow cytometry data of K562 transduced with these vectors show expression of the SIV Env proteins at the cell surface. (D) Representative CellTrace Violet profiles used to analyze the proliferation of CAR T cells as shown in (E). CAR T cells were labeled with CellTrace Violet and incubated in media without (negative control) or with (positive control) the ImmunoCult CD2/3/28 T cell activator. Cells were analyzed 4 days later by flow cytometry and gated on EGFR+ cells before gating on CellTrace Violet. (E) Stimulation of proliferation of anti-SIV CAR T cells by K562 expressing viral envelope or SIV-infected CD4^+^ T cells. CAR T cells labeled with CellTrace Violet were incubated with mitomycin-treated CD4^+^ T cells, SIV-infected CD4^+^ T cells, K562, K562 expressing the full extracellular domain, or the V1V2 region of the SIV Env at a 1 stimulator:1 effector cell ratio. Proliferation was assessed as the percentage of cells present in peaks 3 to 7 corresponding to 2 or more cell divisions as illustrated in (D).

To select an anti-SIV CAR for *in vivo* studies, we compared the potency of CARs based on high-affinity ITS01, ITS06.01, and ITS52 mAbs (Figure 2A). We used labeled SIVmac239 gp140 to confirm the Env binding capability of CARs expressed in transduced CD8^+^ T cells (Figure 2A). Both ITS06 and ITS52 CAR T cells efficiently killed SIVGFP-infected CD4^+^ T cells, with ITS06 CAR T cells being slightly more potent. There was no significant difference between EGFR control T cells and ITS01 CAR T cells in killing targets (Figure 2B).

Several studies have shown the importance of CAR T cell clonal diversity in driving their *in vivo* proliferation.^26^^,^^27^ Because HIV- and SIV-infected T cells express far lower amounts of antigen than that seen in CD19^+^ B cell malignancies, methods to increase antigen expression post-infusion have been proposed. Previous studies showed that infusion of irradiated K562 cells expressing HIV Env 2 weeks after CD4CAR T cell infusion improve expansion of virus-specific CAR T cells *in vivo*.^10^^,^^13^ We chose to adopt a similar strategy by developing antigen-presenting cells (APCs) expressing the SIV Env. K562 cells were transduced with a lentiviral vector containing the full extracellular domain of SIVmac239 Env but cleaved at glycine 720 before the endocytosis motifs that impair surface expression of Env resulting in deletion of amino acids 721 to 879 (K562-Env [del])^28^ and stable surface expression of SIV Env. Because ITS06 targets a linear epitope of the V1 loop, we also generated K562 expressing a V1V2 region of SIV Env (K562-V1V2-Env). To identify these cells, we included the RQR8 marker, since the ITS06 antibody would react with both K562-V1V2-Env and SIV-infected CD4^+^ T cells in blood and tissue biopsies (Figure 2C).^29^ ITS01, ITS06, and ITS52 CAR T cells labeled with CellTrace Violet proliferated following stimulation with K562-Env(del), and ITS06 additionally proliferated when stimulated with K562-V1V2-Env (Figures 2D and 2E). ITS06 and ITS52, but not ITS01, CAR T cells also proliferated when stimulated with SIV-infected CD4^+^ T cells (Figure 2E). Together, these *in vitro* data indicate that the ITS06 CAR T cells are not only highly potent at killing SIV-infected CD4^+^ T cells *in vitro* but also proliferate well when stimulated with Env-expressing K562 and SIV-infected CD4^+^ T cells.

### ITS06 CAR T cells failed to expand after infusion in ART-suppressed SIV-infected RMs

**Figure 3:**
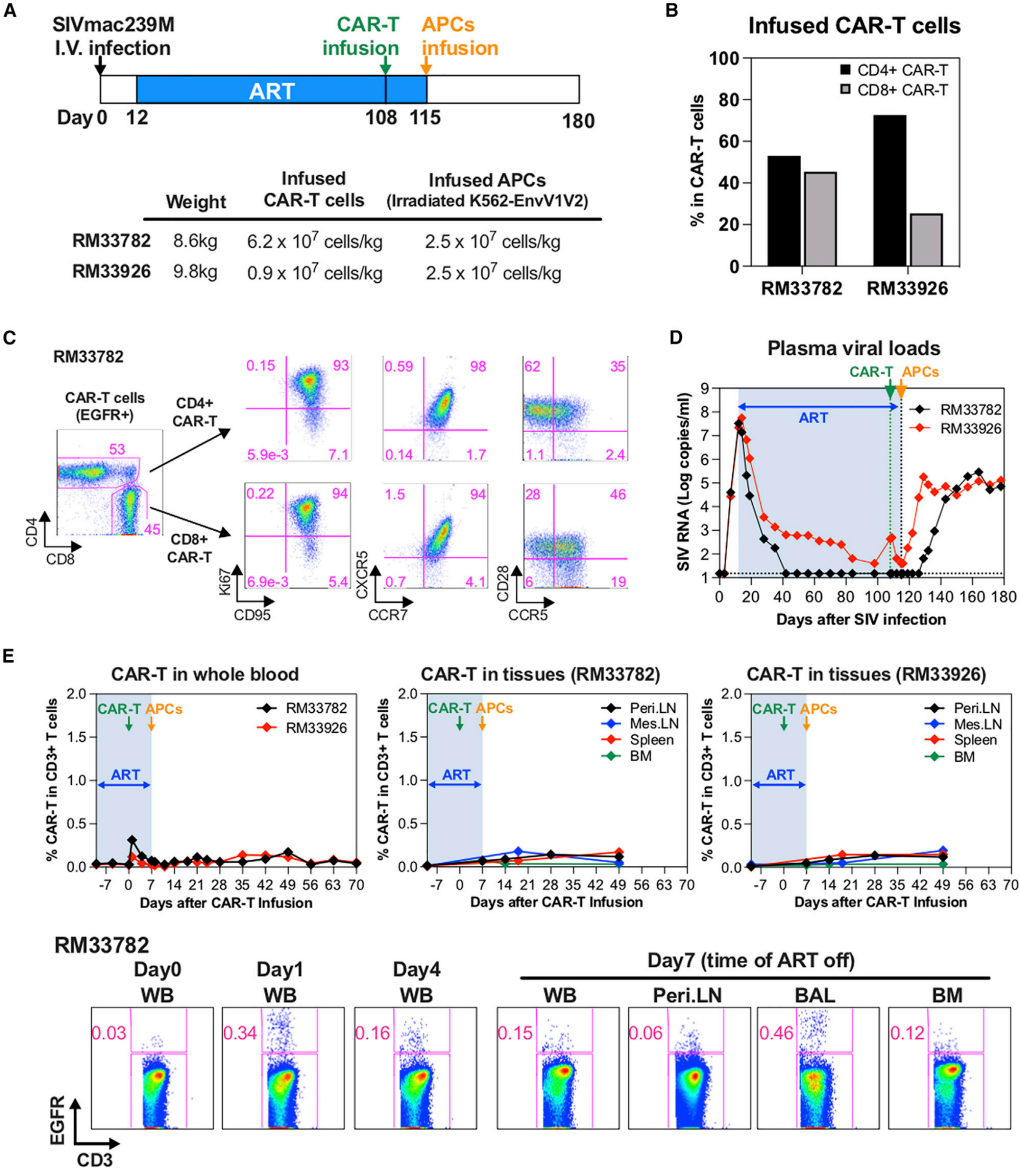
Adoptive transfer of ITS06 CAR T cells in ART-suppressed rhesus macaques (RMs) and their dynamics *in vivo* (A) Schematic representation of the animal study protocol showing SIVmac239M infection (i.v., 500 ffu per RM) and number of injected CAR T and APCs. (B) Ratio of CD4^+^ and CD8^+^ CAR T cells (CD3^+^EGFR^+^) cultured *in vitro* before infusion. (C) Phenotypic characteristics of injected ITS06 CAR T cells (shown for RM33782). (D) Plasma viral load profiles. (E) Top: Frequency of (EGFR^+^) CAR T cells in CD3^+^ T cells in the peripheral blood and tissues after infusion. Bottom: Representative data of CAR T cells in peripheral blood and tissues after infusion (shown for RM33782).

To investigate if ITS06 CAR T cells are capable of persisting *in vivo* and able to delay or reduce viral rebound following antiretroviral therapy (ART) interruption (ATI), we infused CAR T cell products into SIV-infected RMs on ART. Two RMs (RM33782 and RM33926) were intravenously (i.v.) inoculated with SIVmac239 and placed on ART 12 days later (Figure 3A). After 96 days of ART suppression, RM33782 received 6.2 × 10^7^ CAR T cells/kg, while RM33926 was infused with 9 × 10^6^ cells/kg (Figure 3A). With the goal of boosting ITS06 CAR T cell expansion, K562-Env APCs were infused in both animals 7 days later, coinciding with the day of ATI (Figure 3A). RM33782 had a comparable ratio of autologous CD4^+^ and CD8^+^ (53% and 45%, respectively), while RM33926 had CD4^+^-dominant (73%) infused CAR T cells (Figure 3B) The killing potency of both ITS06 CAR T cell products as well as the proliferation of the CAR T cells in the presence of K562-Env V1V2 APCs were similar to those observed in our *in vitro* preclinical assays as described above (data not shown). Thus, the infused cells exhibited potent *in vitro* killing of SIV-infected targets. The majority of infused CAR T cells were proliferating (Ki67^+^), and over 92% of the cells expressed recombinant CXCR5 (Figure 3C). CD4^+^ CAR T cells had phenotypes that indicated central memory (CD95^+^, CD28^+^, CCR5^−^) or transitional effector memory cells (CD95^+^, CD28^+^, CCR5^+^). Approximately, 20% of CD8^+^ CAR T cells were of CD28^−^ effector phenotype (Figure 3C). Following ATI, RM33782, whose plasma viral load (pvl) was below the limit of detection (15 RNA copies/mL) at the time of CAR T infusion, showed viral rebound 14 days after ART release, while RM33926 with a pvl of 430 RNA copies/mL at time of CAR T infusion had an immediate increase in viremia upon ART release (Figure 3D). In both RMs, CAR T cells were detectable in peripheral blood up to 7 days post-infusion even though their number quickly decreased. Similarly, a low frequency (0.46%) of CAR T cells was detected in bronchoalveolar lavage, but no expansion was observed in any of the lymph nodes, spleen, or bone marrow (Figure 3E). In addition, there was no increase in the persistence and/or expansion of the CAR T cells after infusion of K562-Env APCs in both animals. Collectively, these data indicate that while biologically active T cells were infused into both animals, no *in vivo* proliferation/expansion or antiviral activity was documented.

### Infusion of ITS06 CAR T cells in RMs triggered a humoral immune response to the scFv

**Figure 4:**
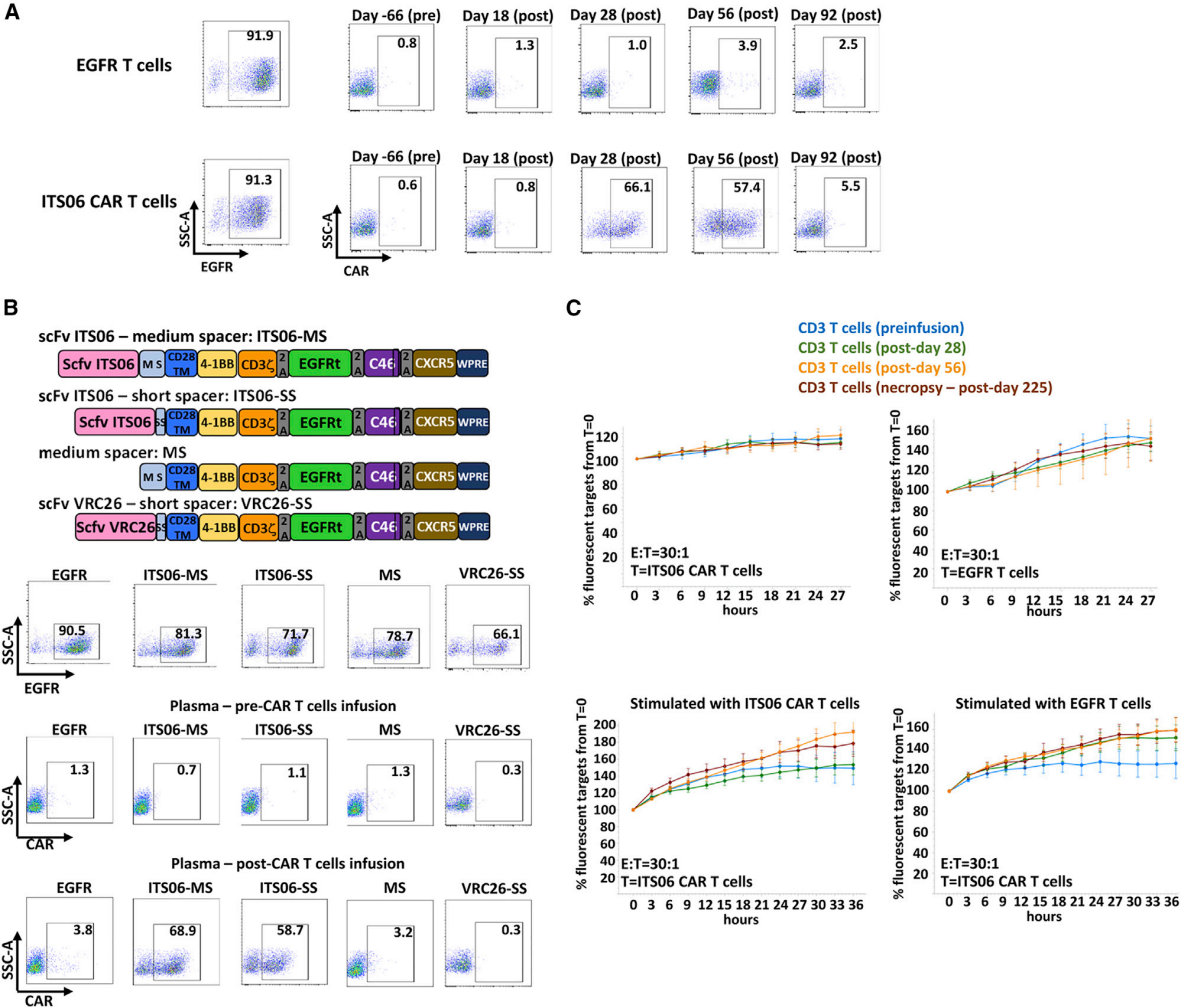
Infusion of autologous ITS06 CAR T cells in RMs triggered an immune response to the scFv (A) Anti-CAR antibodies are detected during a period covering day 28 through day 56 after CAR infusion. Expression of EGFR in ITS06 CAR T cells or EGFR T cells used to probe for anti-CAR antibodies is shown on the left. Plasma of RM33782 collected 66 days before CAR T cell infusion or 18, 28, 56, or 92 days after CAR T cell infusion were incubated with ITS06 CAR T cells or control EGFR T cells. Cell-bound anti-CAR antibodies were then detected by incubation with labeled anti-IgG antibodies. (B) The anti-CAR immune response is directed toward the ITS06 scFv. T cells expressing only the medium spacer (MS) as extracellular domain and as shown on the schematic diagram were generated to assess if antibodies bind to epitope(s) of the 119 amino acid long spacer. Detection of anti-CAR antibodies was also compared between ITS06 CAR including the 119 amino acid medium spacer (ITS06-MS) or the 12 amino acid short spacer (ITS06-SS) or a CAR with a different scFv and the short spacer (VRC26-SS). Plasma of RM33782 collected 66 days before CAR T cell infusion and 56 days after CAR T cell infusion was incubated with T cells transduced with the illustrated CARs or control EGFR T cells followed by incubation with labeled anti-IgG antibodies. (C) Anti-CAR cellular immune response is not detected after infusion of autologous CAR T cells. Fluorescently labeled ITS06 CAR T cells or EGFR control T cells were incubated with CD3 T cells isolated from PBMCs of RM rh33782 collected before infusion or at days 28, 56, or 225 (necropsy) post-infusion and analyzed in the real-time killing assay immediately at thawing (upper panels) or after a 2-week coculture with ITS06 CAR T cells or EGFR T cells (lower panels). The error bars indicate the standard error to the mean.

To investigate possible reasons for lack of proliferation of anti-SIV Env CAR T cells after infusion, we explored whether an immune response against the autologous CAR T cells developed in response to cell infusion. Plasma from animals collected before and after CAR T cell infusion was tested for the presence of antibodies against the CAR by flow cytometry using ITS06 CAR T cells or EGFR T cells as controls. Anti-CAR antibodies were detectable between day 28 and 56 post-infusion for RM33782 infused with 6.2 × 10^7^ cells/kg (Figure 4A). No anti-CAR antibodies were detectable in the plasma of RM33926 that had received 9 × 10^6^ cells/kg. The anti-CAR antibodies in RM33782 were directed against the scFv domain and not the medium spacer because they did not bind to T cells expressing the medium spacer without scFv, yet they reacted with T cells transduced with a CAR containing a short spacer of 12 amino acids (Figure 4B). In addition, the anti-CAR antibodies did not bind to T cells transduced with another CAR built with a similar design but differing only in the sequence of the V_H_ and V_L_ domain that originated from the VRC26.25 mAb, a broadly neutralizing antibody targeting the V1V2 region of HIV-1 Env (Figure 4B).^30^ We also tested if a cellular immune response had been induced post-infusion against the autologous anti-ENV CAR T cells. CD3 T cells were isolated from peripheral blood mononuclear cells (PBMCs) collected preinfusion and at day 28, 56, and 225 post-infusion and mixed with autologous ITS06 CAR T cells or EGFR T cells. No killing of ITS06 CAR T cells or control EGFR T cells was detectable (Figure 4C, upper panels). To enrich putative anti-CAR T cells that might have been at low levels, the CD3 T cells isolated from RM33782 were cocultured for 2 weeks with autologous ITS06 CAR T cells and retested in the killing assay. Still, no cell-mediated killing of CAR T cells was detectable (Figure 4C, bottom panels).

### Anti-SIVmac239 Env antibodies inhibit killing of SIV-infected CD4^+^ T cells by ITS06 CAR T cells

**Figure 5:**
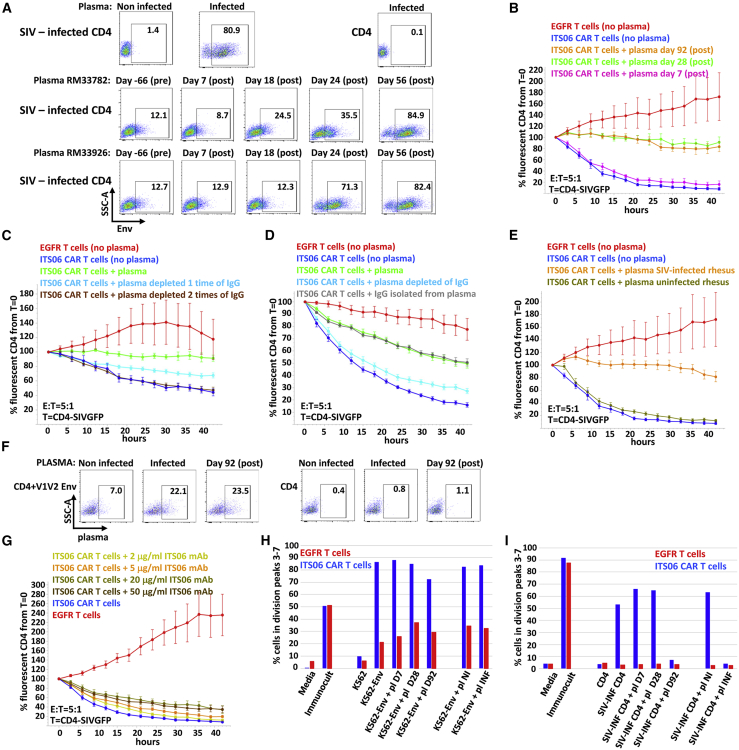
Anti-Env antibodies inhibit killing of SIV-infected CD4^+^ T cells by ITS06 CAR T cells (A) Anti-Env antibodies are detected at all time points in SIV-infected RMs. Plasma collected as indicated or from noninfected or SIV-infected animals was incubated with SIV-infected CD4^+^ or CD4^+^ T cells. Cell-bound antibodies were detected with labeled anti-IgG antibodies. (B) A 20× dilution of the plasma from RM33782 collected at day 28 or 92 post-infusion inhibits killing of SIV-infected targets by the CAR T cells. The error bars indicate the standard error to the mean. (C) Reversal of this inhibition after IgG removal. Plasma collected at day 92 post-infusion was incubated once or twice with protein G-coated beads and then added to the killing assay. (D) IgGs are responsible for this inhibition. After incubation of the plasma collected at day 92 post-infusion with protein G beads, the IgGs were eluted from the beads and added to the assay. (E) Anti-Env antibodies inhibit killing of SIV-infected targets by CAR T cells. Plasma from SIV-infected animals that have not been infused with CAR T cells inhibits killing of SIVGFP-infected targets by CAR T cells. A control with plasma from an uninfected animal does not show any inhibition effect. Representative data for plasma of 2 animals each are shown. (F) Antibodies reacting with epitopes present in the V1V2 region targeted by the ITS06 CAR are present in the plasma from SIV-infected animals infused or not with CAR T cells. (G) ITS06 mAbs have minimal effect on CAR T cell effector function. (H) Proliferation of the CAR T cells in response to K562-Env APCs is not inhibited in the presence of a 10× dilution of the plasma of SIV-infected animals (pl, plasma; D, day; INF, infected; NI, non-infected). (I) Inhibition of proliferation of the CAR T cells in response to SIV-infected CD4^+^ T cells in the presence of a 10× dilution of the plasma of SIV-infected animals (pl, plasma; D, day; INF, infected; NI, non-infected).

We next investigated whether lack of expansion of ITS06 CAR T cells *in vivo* might be partially due to the presence of anti-Env antibodies masking the epitope targeted by the CAR T cells. First, we confirmed the presence of anti-Env antibodies in the plasma of SIV-infected animals during ART and after ATI using flow cytometry. Although the plasma from ART-treated SIV-infected RMs collected before CAR T cell infusion was less reactive against SIV-infected CD4^+^ T cells than plasma collected a few weeks after ATI and CAR T cell infusion, anti-Env antibodies were detectable in all samples (Figure 5A). In addition, even if RM33926 was not fully suppressed at the time of the infusion (Figure 3D), the plasma from both animals showed similar reactivity against infected cells (Figure 5A). We tested if antibodies present in the plasma might block access to the epitope targeted by the ITS06 CAR and thus interfere with killing of SIVGFP-infected CD4^+^ T cells. Plasma collected 3 weeks after ATI and at later time points inhibited CAR T cell killing of targets (Figure 5B). Plasma IgG depletion using protein G resulted in a loss of inhibition demonstrating that the inhibitory activity was mediated by IgG subclass antibodies (Figure 5C). Moreover, IgGs purified from RM33782 post-infusion plasma were as effective as the plasma in blocking killing of SIV-infected CD4^+^ T cells (Figure 5D). Because anti-Env as well as anti-CAR antibodies could block epitope recognition, we assessed the effect of heterologous plasma from uninfused SIV-infected animals. A similar decrease in killing potency mediated by CAR T cells was observed in the presence of plasma from infected animals but not with heterologous plasma from uninfected animals (Figure 5E). These data indicate that anti-Env antibodies are sufficient in blocking target recognition. We also tested whether antibodies binding to the V1V2 domain were detected in the plasma of SIV-infected animals. Antibodies reacting with CD4^+^ T cells transduced with the lentiviral vector encoding the V1V2 Env peptide were detected using flow cytometry (Figure 5F). Nevertheless, it is likely that many diverse antibodies reacting with the SIV envelope hinder the accessibility of the V1 epitope to the CAR because ITS06, at concentration as high as 50 μg/mL, only slightly interferes with the ITS06 CAR T cell-mediated killing of SIV-infected cells (Figure 5G). Plasma collected from the infused animals and from heterologous infected animals also inhibited the proliferation of ITS06 CAR T cells in response to SIV-infected CD4^+^ T cells but not in response to K562-Env cells (Figures 5H and 5I). Together, these data suggest that anti-Env antibodies interfere with recognition by CAR T cells of Env antigens present on SIV-infected cells.

### Effect of anti-Env antibodies on the potency of other anti-SIV Env CAR T cells

**Figure 6:**
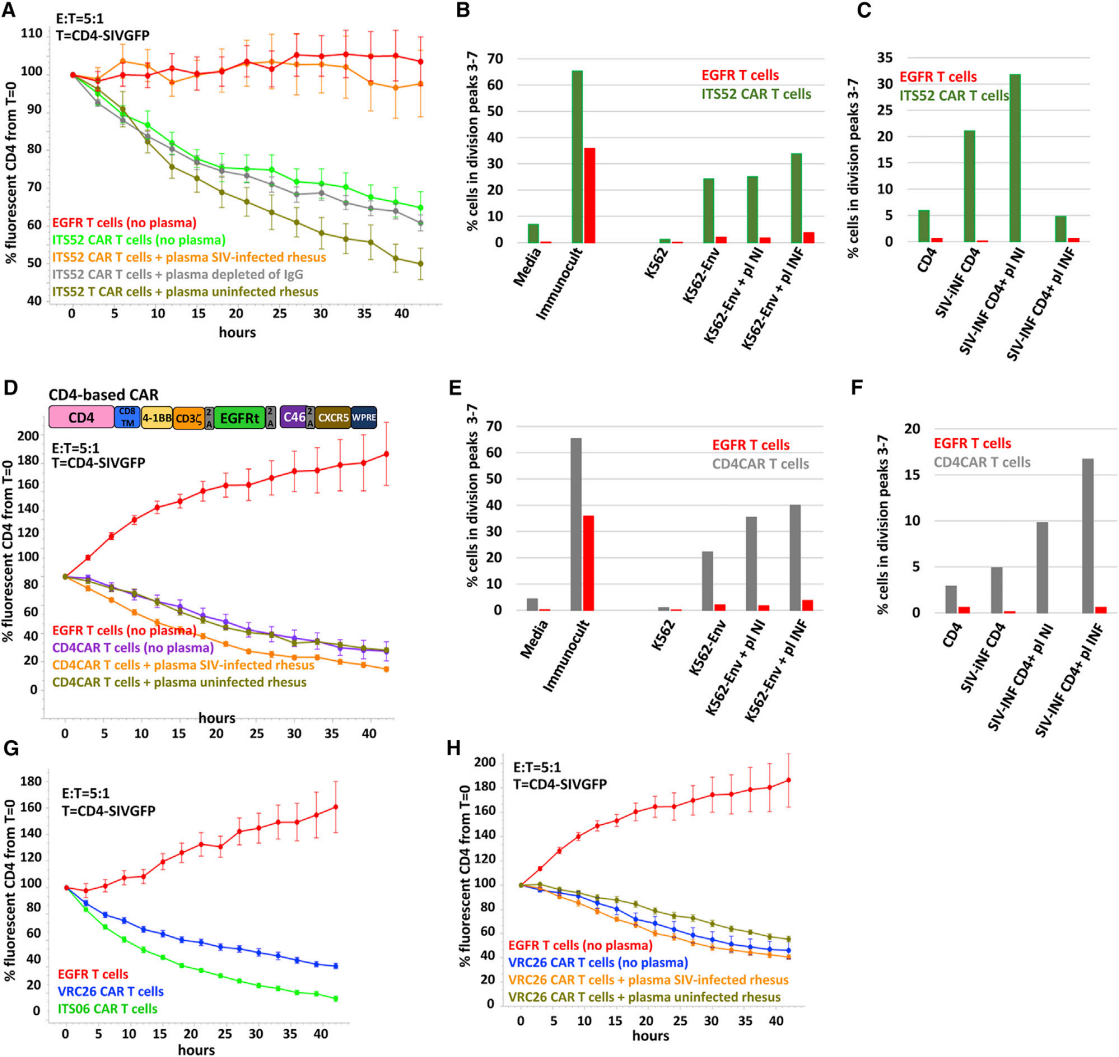
Effect of anti-Env antibodies on the potency of other anti-SIV Env CAR T cells (A) The potency of ITS52 CAR T cells is inhibited by anti-Env antibodies. ITS52 CAR T cells and SIVGFP-infected CD4^+^ T cells were mixed in the presence of a 20× dilution of plasma of uninfected or SIV-infected RMs and analyzed in a real-time killing assay. The error bars indicate the standard error to the mean. (B) Stimulation of proliferation of the ITS52 CAR T cells by K562-Env is not affected in the presence of a 10× dilution of plasma from SIV-infected animals. (C) Plasma from SIV-infected animals inhibit stimulation of proliferation of the ITS52 CAR T cells by SIV-infected CD4^+^ T cells. (D) No inhibition of the potency of CD4-based CAR T in the presence of anti-Env antibodies. A 20× dilution of plasma of uninfected or SIV-infected RMs was added to a mix of CD4CAR T cells and SIVGFP-infected targets. (E) Proliferation of CD4CAR T cells is similar in the presence of plasma from uninfected and SIV-infected animals (pl, plasma; D, day; INF, infected; NI, non-infected). (F) Stimulation of proliferation of the CD4CAR T cells by SIV-infected CD4^+^ T cells is not inhibited by the plasma from SIV-infected animals (pl, plasma; D, day; INF, infected; NI, non-infected). (G) VRC26 CAR T cells (see VRC26-SS in Figure 4B) are cytotoxic toward SIVGFP-infected cells but are less potent killers than ITS06 CAR T cells. (H) No inhibition of the killing potency of VRC26 CAR T in the presence of anti-Env antibodies. A 20× dilution of plasma was added to the assay when indicated.

To investigate if the anti-Env antibodies naturally occurring in response to SIV infection would interfere with the potency of other anti-Env CAR T cells, we tested the effect of adding plasma from SIV-infected animals to ITS52 CAR T cells. Anti-Env antibodies inhibited killing of SIVGFP-infected CD4^+^ T cells by ITS52 CAR T cells (Figure 6A). Plasma of SIV-infected animals also interfered with the stimulation of ITS52 CAR T cells by SIV-infected CD4^+^ T cells but not by K562-Env cells (Figures 6B and 6C). Another type of anti-Env CAR is the CD4CAR based on the CD4 receptor that interacts with viral gp120 during infection.^13^ Because CD4-based CAR T cells have been shown to successfully expand *in vivo* after infusion in SHIV-infected RMs, we also tested whether their potency would be affected by anti-Env antibodies.^13^ Plasma from SIV-infected animals had no effect on the killing potency of CD4CAR T cells toward SIVGFP-infected CD4^+^ T cells (Figure 6D). Proliferation of CD4CAR T cells in response to stimulation by SIV-infected CD4^+^ T cells or K562-Env cells was also not inhibited in the presence of plasma from SIV-infected animals (Figures 6E and 6F). These data suggest that anti-Env CAR T cells whose potency and proliferation are not affected by anti-Env antibodies might persist longer after infusion. As part of another study, we also screened other anti-Env CAR T cells based on the scFv of HIV-1 bnAbs for their potency in killing SHIV-infected CD4+ T cells.^31^ The scFv of VRC 26 directed at the V2 loop of HIV-1 was isolated from an HIV-1 infected donor.^30^ VRC26 CAR T cells killed SIV-infected cells (Figure 6G), albeit less efficiently than ITSO6 CAR T cells. VRC26 CAR T cell-mediated killing of SIV-infected cells was not inhibited by plasma from SIV-infected animals (Figure 6H).

## Discussion

Our study reveals several findings about the choice of targets for CAR T cells, especially to infectious disease antigens. Our findings indicate that high titer of antibodies directed at the CAR T cell receptor binding site can abrogate *in vivo* proliferation and killing by the CAR T cell. Highly potent CAR T cells to the V1 and V3 loops were constructed with strong *in vitro* killing of target cells. Yet, *in vivo*, neither proliferation nor expansion was noted despite infusion of functionally active cells. We discovered that antibodies to the V1 and V3 regions were present in sera and that plasma from uninfected animals did not inhibit proliferation of CAR T cells *in vitro*, but purified IgG to SIV Env from plasma of infected animals inhibited CAR T cell-mediated killing. Thus, endogenous antibodies appeared to abrogate the clonal activity of the construct. Our data raise the issue of whether this *in vivo* elicitation of antibodies will be observed with other CAR T cell programs and pathogens.

Our studies emanated from the observation that ITS06 CAR T cells exhibited excellent *in vitro* killing and proliferation at the time of infusion but did not expand *in vivo* with antigen stimulation. First, we detected a humoral immune response against the scFv domain of the CAR in RM33782 infused with 6.2 × 10^7^ CAR T cells/kg. Second, we also discovered that the natural immune response to SIV infection alone, independently of the anti-CAR immune response, interfered with CAR T cell-mediated killing of SIV-infected CD4^+^ T cells. Because the anti-CAR immune response was detected only in RM33782 and 3 weeks after the infusion of the CAR T cells, but lack of expansion occurred in both animals, our data suggest that it is more likely the immune response induced by viral infection that resulted in poor persistence of the CAR T cells. Indeed, we also demonstrated that plasma collected from other SIV-infected RMs that were not infused with CAR T cells were equally detrimental to CAR T cell killing activity. In addition, immune responses to various CARs have been observed in human clinical studies where expansion of the CAR T cells was observed.^32^, ^33^, ^34^, ^35^, ^36^, ^37^, ^38^

Although antibodies directed against the V1V2 region were detected in the plasma of the infused animals, the effect of purified ITS06 mAbs on the CAR T cell effector functions was less than that of antibodies from plasma, suggesting that antibodies reacting with other domains of SIV Env sterically hindered access of the anti-Env CAR T cells to the viral protein. While the functionality of ITS52 CAR T cells was also affected by the plasma of SIV-infected animals, the potency of CD4CAR T cells was not affected and is consistent with data showing successful expansion of CD4CAR T cells *in vivo* after infusion in SHIV-infected RMs.^13^ Although isolated from HIV-infected patients, we discovered that the scFv-based VRC26 CAR T cells can also kill SIV-infected cells. The killing activity of the VRC26 CAR T cells was not inhibited by the plasma from SIV-infected animals. This result implies that (1) the host immune response generated during SIV infection does not block neutralizing antibodies induced during infection with other immunodeficiency viruses, and (2) the interference of the infection-induced antibodies with the functionality of a CAR is not general to all scFv-based CARs and that VRC26 CAR might be a better candidate for future preclinical studies.

CAR T cell proliferation in response to stimulation by SIV-infected CD4^+^ T cells was also inhibited by the plasma of SIV-infected RMs. Surprisingly, plasma from infected animals had no effect on CAR T cell proliferation in response to stimuli by K562-Env. At this point, the causes for the different effect of plasma in inhibiting proliferative stimuli mediated by K562-Env or SIV-infected CD4^+^ T cells is not known. A difference between both APCs in the density of envelope proteins on the cell membrane might influence the ability of the plasma antibodies to interfere with antigen recognition. Although our data showed that anti-Env antibodies do not interfere with proliferative stimuli by K562-Env cells, these APCs did not boost the expansion of CAR T cells *in vivo*. It is possible that the frequency of CAR T cells had already declined below a threshold necessary for sufficient expansion at the time of K562-Env infusion and it was too late for APCs to trigger expansion.

The detrimental effect of the antibody response to the CAR or viral antigens could limit the use of scFv-based CAR T cells as a therapy to cure HIV unless strategies are developed to circumvent it. One possibility to reduce anti-CAR immune response would be to induce B cell depletion by infusion of antibodies targeting B cells prior to CAR T cell infusion. Indeed, anti-CD20 and anti-CD19 therapy directly targeting B cells has been used for the treatment of autoimmune disease and led to reduced levels of B cells and serum antibodies.^39^^,^^40^ Another strategy would be to map the epitope(s) of the CAR that triggered the anti-CAR humoral response. We could then modify this epitope(s) while keeping the binding ability of the CAR or develop anti-idiotypic antibodies that could be injected with the CAR T cells. In addition, before initiating *in vivo* studies, the selection of anti-viral CAR T cells *in vitro* should be performed in the presence or absence of plasma of infected animals to determine if anti-viral antibodies might affect their killing potency. CD4CAR T cells are an example of a good candidate, because their killing activity against SIV-infected cells is not affected by anti-viral antibodies.

Optimized cell manufacturing is essential to increase the persistence and expansion of CAR T cells *in vivo*.^41^^,^^42^ Because our CAR T cell infusion product was prepared using the same manufacturing protocol that had been developed previously for a successful *in vivo* experiment,^13^ it is unlikely that the lack of expansion of ITS06 CAR T cells was due to the quality of the infused product. As expected, our characterization of the CAR T cells in the infusion product exhibited a central memory and effector memory phenotype shown to be important for the long-term persistence of the cells *in vivo*.^20^^,^^43^^,^^44^ In addition, before initiating the *in vivo* analysis of ITS06 CAR T cells, we optimized the design of our CAR lentiviral vector. In particular, it was crucial to protect the CD4^+^ ITS06 CAR T cells against SIV infection and thus also CAR T fratricide, since CD4^+^ CAR T cells are as efficient in killing SIV-infected cells as CD8^+^ CAR T cells and have been shown to be critical in maintaining CD8^+^ T cell function.^19^^,^^20^ Because our data showed that co-expression of the gp41-derived C46 fusion inhibitor provided protection against SIV infection in up to 85% of the ITS06 CAR T cells *in vitro*, it is unlikely that infection of CD4^+^ CAR T cells was a major contributor to their short *in vivo* persistence.

In conclusion, despite the optimization of our CAR T cell design and use of a proven CAR T cell manufacturing protocol, potent anti-Env CAR T cells had very limited persistence *in vivo* after transfer in RMs. This study revealed possible obstacles to the successful adoptive transfer of CAR T cells: an anti-scFv immune response against the antibody-based CAR and the presence of epitope blocking antibodies that interfere with CAR T cell functionality. Immune response against CAR T cells is known as a possible cause for the elimination of infused CAR T cells, but our study showed that anti-viral antibodies add additional difficulties to the success of CAR T cell therapy to treat viral diseases. Although the extent to which the anti-Env and anti-CAR antibodies could interfere with CAR T cell persistence has not been assessed *in vivo*, they likely impaired target recognition and subsequently CAR T cell activation and proliferation. In view of these observations, addressing these issues during *in vitro* characterization and selection of other CARs as well as developing strategies and optimizing lymphodepletion regimens to reduce anti-CAR immune responses could improve the success rate of CAR T cell adoptive transfer for diseases in which host immune responses to the scFv are likely to be present.

## Materials and methods

### Generation of lentiviral transfer plasmids

The configuration and potency of all vectors are summarized in Table 1. The ITS06 CARs were generated by linking in both configurations with a (G_4_S)_3_ peptide the V_H_ and V_L_ domains corresponding to the ITS06.01 mAb that targets an epitope in the V1 loop of the SIV envelope.^2^ This ScFv was also connected to the CD28 transmembrane domain using spacers of different lengths: a short spacer (human IgG4 hinge of 12 amino acids), a medium spacer (human IgG4 hinge-CH3 of 119 amino acids), and a long spacer (human IgG4 hinge-CH2-CH3 of 228 amino acids). For all CARs, the intracellular domain is composed of a 4-1BB intracellular costimulatory domain and a CD3ζ signaling domain and is fused to a truncated EGFR as a marker to identify transduced cells via a Thosea asigna virus 2A (T2A) self-cleavage peptide. These CAR-EGFRs or EGFRs were then cloned together with a woodchuck hepatitis virus posttranscriptional regulatory element (WPRE) into a SIV-based lentiviral vector (a generous gift from Dr. Nienhuis, St. Jude Children’s Research Hospital, Memphis, TN and Dr. Miyazaki, Osaka University, Japan).^45^^,^^46^ These elements were cloned between the AgeI and NotI site of the pCL20c MSCV-GFP plasmid downstream of the murine stem cell virus (MSCV) promoter using the NEBuilder HiFi DNA Assembly (New England Biolabs). The production of recombinant lentiviruses was performed as previously described.^18^ Briefly, Lenti-XTM 293T cells (Takara Bio) in DMEM media containing 10% fetal bovine serum (FBS) and 100 U/mL Pen/Strep were transfected using the standard calcium phosphate method with 15 μg of the CAR transfer vector together with 6 μg of the pCAG-SIVgprre plasmid carrying the *gag/pol* and *rev* responsive element (RRE), 4 μg of the *rev/tat* expression plasmid pCAG4-RTR-SIV, and 3 μg of the pMD2.CocalG containing the glycoprotein G of the cocal virus.^47^ The next day, fresh media was added after washing the HEK293T cells with phosphate-buffered saline (PBS). One and 2 days later, lentivirus-containing media was collected, cleared by centrifugation at 1,000 × *g* for 5 min followed by filtration on a 0.45 μm Millipore filter, and concentrated by ultracentrifugation at 74,000 × *g* for 2 h at 4°C. The lentivirus stocks (concentrated, 1:50 in PBS) were then titrated by transduction of Jurkat cells cultured in RPMI media supplemented with 10% FBS and 100 U/mL Pen/Strep with 4 μg/mL polybrene followed by spinoculation for 2 h at 1,200 × *g*. The percentage of transduced Jurkat cells was quantified by flow analysis for EGFR using the anti-EGFR cetuximab mAb (Erbitux, PE-conjugated at Juno Therapeutics).

**Table 1:** Plasmid DNA vectors generated in this study and their killing activity

	scFv	Short spacer (12 AA)	Medium spacer (119 AA)	Long spacer (228 AA)	CD28 TM	4-1BB	CD3ζ	EGFRt	C46	CXCR5	Killing activity
EGFRt								✓			
V_H_-V_L_-SS (ITS06)	V_H_-V_L_	✓			✓	✓	✓	✓			✓
V_H_-V_L_-MS (ITS06)	V_H_-V_L_		✓		✓	✓	✓	✓			✓
V_H_-V_L_-LS (ITS06)	V_H_-V_L_			✓	✓	✓	✓	✓			✓
V_L_-V_H_-SS (ITS06)	V_L_-V_H_	✓			✓	✓	✓	✓			✓
V_L_-V_H_-MS (ITS06)	V_L_-V_H_		✓		✓	✓	✓	✓			✓
V_L_-V_H_-LS (ITS06)	V_L_-V_H_			✓	✓	✓	✓	✓			✓
ITS06-SS	V_H_-V_L_	✓			✓	✓	✓	✓	✓	✓	✓
ITS06-MS	V_H_-V_L_		✓		✓	✓	✓	✓	✓	✓	✓
MS			✓		✓	✓	✓	✓	✓	✓	
VRC26-SS	V_H_-V_L_	✓			✓	✓	✓	✓	✓	✓	✓
ITS06 CAR del CD3ζ	V_H_-V_L_		✓		✓	✓		✓			
ITS06 CAR del CD3ζ-C46	V_H_-V_L_		✓		✓	✓		✓	✓		
ITS01 CAR	V_H_-V_L_	✓			✓	✓	✓	✓	✓	✓	
ITS06 CAR	V_H_-V_L_		✓		✓	✓	✓	✓	✓	✓	✓
ITS52 CAR	V_H_-V_L_	✓			✓	✓	✓	✓	✓	✓	✓
EGFR								✓	✓	✓	
CD4CAR	CD4 receptor—CD8 TM					✓	✓	✓	✓	✓	✓

	SIV Env (AA 1–720)	SIV Env (AA 78–232)	RQR8								

SIV Env (del)	✓										
V1V2-Env		✓	✓								

AA, amino acid; TM, transmembrane domain.

All the following clonings were performed using the NEBuilder HiFi DNA Assembly. To generate a SIV Env CAR-EGFR-C46 vector, the C46 inhibitory peptide preceded by a T2A self-cleavage peptide and a signal peptide and linked through an IgG2 hinge to the membrane spanning domain of CD34^48^ was first added as a PCR product to the ITS06-V_H_-V_L_-MS-CAR construct. A cDNA encoding the rhesus CXCR5 (SinoBiological) preceded by a porcine teschovirus-1 2A (P2A) cleavage site was then added downstream of the SIV Env CAR-EGFR-C46 to build the SIV Env CAR-EGFR-C46-CXCR5. The CD3ζ domain was deleted from the SIV Env CAR-EGFR-C46 and SIV Env CAR-EGFR to create the SIV Env CAR-delCD3ζ-EGFR-C46 and SIV Env CAR-delCD3ζ-EGFR, respectively.

The medium spacer plasmid was constructed by deleting the scFv present between the IgG kappa signal peptide and the medium spacer in the SIV Env CAR-EGFR-C46-CXCR5 lentiviral plasmid. The ITS06 V_H_-V_L_ CAR with the short spacer described above was switched with the ITS06 V_H_-V_L_-MS in the SIV Env CAR-EGFR-C46-CXCR5 plasmid to create the ITS06-SS plasmid.

The ITS01 CAR and ITS52 vectors were generated by switching the scFv in the SIV Env CAR-EGFR-C46-CXCR5 plasmid with the scFv of the ITS01 or ITS52 mAb that targets epitopes in the CD4 binding site or V3 loop of the SIV Env, respectively.^2^ The VRC26 CAR vector was generated by switching the scFv in the ITS06-SS plasmid with the scFv of the VRC26.25 bnAb.^30^

To generate the SIV Env del 721-879 plasmid, a DNA fragment covering amino acids 1–720 of the SIVmav239 followed by a stop codon was cloned between the MSCV promoter and the WPRE of the lentiviral vector described above. To build the V1V2-SIV Env plasmid, a region encompassing the V1V2 domain (amino acids 78–232) of the SIV-env was cloned with the same design as the C46 peptide after the signal peptide and before the hinge and membrane-spanning domain. This V1V2 domain, together with a DNA fragment encoding a P2A cleavage site fused to the RQR8 marker^29^ that was synthesized (Integrated DNA Technologies), was cloned between the MSCV promoter and the WPRE of the lentiviral vector.

### Preparation and transduction of CD4^+^ and CD8^+^ RM T lymphocytes for the *in vitro* experiments

The preparation and transduction of T lymphocytes from frozen PBMCs from RMs (*Macaca mulatta*) and their analysis by flow cytometry were performed as previously described.^18^ Briefly, CD4^+^ and CD8^+^ T cells were enriched from PBMCs by negative selection using immunomagnetic beads (Easy Sep nonhuman primate [NHP], STEMCELL Technologies). T cells were cultured in X-vivo 15 media (Lonza) including 10% FBS, 100 U/mL Pen/Strep, 1× glutamax, 50 μM β-mercaptoethanol, 50 IU/mL human recombinant IL-2, 5 ng/mL of human interleukin-7 (IL-7), and 5 ng/mL IL-15 (PeproTech) at 37°C in a humidified 5% CO_2_ atmosphere. Expansion of T cells was triggered by the addition of ImmunoCult NHP T cell activator (STEMCELL Technologies). After 3 days, CD4^+^ and CD8^+^ T cells mixed at a ratio of about 1:1 were transduced with CAR lentivirus at a multiplicity of infection (MOI) of ~2 using spinoculation for 2 h at 1,200 × *g*. The next day, cells were washed and expanded in fresh media. Four days later, T cells were analyzed by flow cytometry for cell surface protein expression using PE-anti-EGFR, BV421 anti-CD4 (OKT4, Biolegend), APC-Cyanine7 anti-CD8 (SK1, Biolegend), and biotinylated anti-CXCR5 (MU5UBEE, eBioscience) combined with BV421 Streptavidin (Bioscience). Receptor expression was analyzed using FlowJo after sequential gating on lymphocytes and single cells. For the detection of cell-bound SIVmac239 gp140 (Immune Technology) after incubation with CD8^+^ CAR T cells, SIV-Env-specific ITS06 or ITS52 mAb^2^ biotinylated with EZ-Link Sulfo-NHS-LC-Biotin (Thermo Scientific) was added to the cells followed by incubation with BV421 streptavidin (Biolegend) and analysis using flow cytometry.

### Real-time killing assay

The preparation of EGFP SIVmac239 viruses (SIVGFP) and fluorescent targets and the real-time killing assay were performed as previously described. Briefly, 2 days after transfection of HEK293T cells with 20 μg of the SIVmac239-GFP, the cell supernatant was filtered on a 0.45 μm Millipore filter and the virus was concentrated using lenti-X concentrator (Takara Bio) following the manufacturer’s instructions. Aliquots of the SIV-EGFP virus (concentrated, 1:25 in PBS) were stored at −80°C. Infection of CD4^+^ T cells was performed by adding 20 μL of concentrated SIVGFP viruses to 10^5^ CD4^+^ T cells (~MOI: 0.5) plated on retronectin-coated 96-well plates followed by spinoculation for 2 h at 1,200 × *g* and incubation for ~3 days at 37°C.

Fluorescent SIV-GFP-infected CD4^+^ T cells were mixed with effector CAR T cells at various effector-to-target (E:T) ratios as indicated. Plates were incubated at 37°C in the IncuCyte S3 LiveCell Analysis System (Sartorius), and five images of each triplicate of wells were recorded every 3 h and analyzed with the IncuCyte image analysis software. The killing potency of the anti-SIV CAR T cells was assessed by comparing the number of fluorescent targets over time relative to their number at T = 0. The error bars indicate the standard error to the mean. When indicated, various concentrations of purified ITS06 mAbs or a final 1:20 dilution of plasma collected from RM and heat inactivated for 30 min at 56°C was added to the assay at T = 0. For some experiments, plasma was depleted of IgG by incubation with protein G magnetic beads (Sigma-Aldrich) following the manufacturer’s protocol. After removal of the beads with a magnet, the plasma was added to the killing assay at a final dilution matching the untreated plasma. IgGs were occasionally eluted from the beads and dialyzed against PBS before addition to the killing assay.

### Analysis of inhibition of SIVmac239 infection in C46-expressing T cells

Seven days after transduction with the ITS06 CAR deleted of the CD3ζ domain with or without the C46 fusion inhibitor, CD4^+^ T cells were infected with SIVmac239 by spinoculation as described above and incubation overnight at 37°C. The next day, cells were washed, fresh media was added, and cells were cultured for another 2 days. The percentage of SIVGFP-infected cells was assessed using flow cytometry after gating on lymphocytes and single cells. Protection was assessed by comparing the percentage of GFP-expressing cells among transduced EGFR+ cells and untransduced EGFR− cells.

### Generation of K562-Env APCs and assessment of CAR T cell proliferation

K562 cells (ATCC, CCL-243) were transduced with a lentivirus vector encoding the extracellular and transmembrane domain of the SIVmac239 Env (amino acids 1–720) or a V1V2 domain region of the SIV Env (amino acids 78–232). Envelope expression was evaluated using flow cytometry and staining with a biotinylated ITS06.01 antibody followed by BV-421 streptavidin and/or an anti-RQR8/CD34 (Qbend/10, R&D Systems) for the K562-V1V2 Env. Transduced K562 cells were isolated using magnetic-activated cell sorting (MACS) (Miltenyi Biotec) following the manufacturer’s instructions after incubation with a biotinylated ITS06.01 antibody.

To assess CAR T cell proliferation in response to stimulators, CellTrace Violet (Thermo Fisher)-labeled CAR T cells or EGFR T cells were cocultured at a ratio of 1 stimulator:1 CAR or EGFR T cell with mitomycin C-treated K562-Env or mitomycin C-treated SIV-infected CD4^+^ T cells or their respective negative control K562 or CD4^+^ T cells for 4 days at 37°C. Cells were harvested and stained with anti-EGFR, and proliferation was assessed by determining the percentage of EGFR+ cells present in peaks 3–7 of decreased CellTrace Violet fluorescence intensity after gating on lymphocytes, single cells, and EGFR+ cells using the FlowJo software.

### CAR T cell manufacturing for the *in vivo* experiments

CAR T cells were prepared as previously described.^13^ Briefly, autologous T cells were isolated from PBMCs collected from the animals before SIV infection by CD4-positive selection, followed by CD8-negative selection using immunomagnetic beads (EasySep nonhuman primate, STEMCELL Technologies). Cells were cultured in X-VIVO-15 media including 50 μM β-mercaptoethanol, 10% FBS, 1% penicillin/streptomycin, 1% GlutaMAX, and 5 ng/mL each of human IL-7 and IL-15. NHP T cells were stimulated with an artificial APC (aAPC) cell line engineered to express CD86 and an anti-CD3 single-chain variable fragment.^49^ These aAPCs were irradiated at a dose of 100 Gy and mixed with NHP T cells at a ratio of 1 aAPC:2 T cells. After 3 days of stimulation, CD4^+^ and CD8^+^ T cell were transduced with lentiviral vector at an MOI of 10. The next day, CD4^+^ and CD8^+^ cells were pooled at a ratio of 1:1 in G-Rex flasks (Wilson Wolf) and expanded for 4 days.

### Infusion of CAR T cells in RMs

These experiments used two RMs (*Macaca mulatta*, both male, Trim5 Q/Q) of Indian genetic background with the approval of the Oregon National Primate Research Center’s Animal Care and Use Committee, under the standards of the US National Institutes of Health Guide for the Care and Use of Laboratory Animals. These RMs were specific pathogen-free as defined by being free of cercopithecine herpesvirus 1, D-type simian retrovirus, simian T-lymphotropic virus type 1, rhesus rhadinovirus, and *M. tuberculosis*. The RMs were i.v. inoculated with 500 TZM-bl assay focus-forming units of RM PBMC-expanded SIVmac239M and placed on ART starting at 12 days post infection and maintained on ART. Prior to SIV infection, leukapheresis was performed periodically (twice per RM), and PBMCs were isolated and frozen for the production of CAR T cells. ART consisted of a subcutaneous injection of 5.1 mg kg^−1^ d^−1^ tenofovir disoproxil, 40 mg kg^−1^ d^−1^ emtricitabine (FTC), and 2.5 mg kg^−1^ d^−1^ dolutegravir in a solution containing 15% (v/v) kleptose at pH 4.2, as previously described.^50^ Each recipient received a single dose of cyclophosphamide (Baxter, 30 mg/kg) for lymphodepletion on day −5 prior to CAR T cell infusion. ITS06 CAR T cells were infused i.v. at doses of 0.9 × 10^7^ or 6.2 × 10^7^ EGFR+ T cells/kg. On day 7 post CAR T infusion, ART was stopped, and the APCs (irradiated K562-V1V2 Env) were given i.v. to the monkeys at a dose of 2.5 × 10^7^ cells/kg. Whole blood, peripheral lymph nodes (Peri.LN), mesenteric lymph nodes (Mes.LN), spleen, bronchoalveolar lavage (BAL), and bone marrow aspirates (BM) were collected longitudinally in all recipients. Recipient RMs are followed for a minimum of 80 days for the onset of plasma viremia.

### Viral detection assays

Plasma SIV RNA levels were determined using a gag-targeted quantitative real-time/digital RT-PCR format assay, essentially as previously described, with 6 replicate reactions analyzed per extracted sample for assay thresholds of 15 SIV RNA copies/mL.^51^

### CAR T cell detection and immunophenotyping

To evaluate the *in vivo* CAR T cells, whole blood and mononuclear cell preparations from tissue biopsies were stained with antibodies labeled with fluorochromes for cytometric analysis. All samples (100 μL whole blood or 10^6^ small lymphocytes from tissue samples or cultured CAR T cells) were initially stained with anti-EGFR (Hu1: Biotin, R&D Systems), and Live/Dead Fixable Aqua Dead Cell Stain Kit (Thermo Fisher) for 30 min. After washing, the samples were stained with surface antibodies 30 min: CD45 (D058-1283: BUV395, BD Biosciences), anti-CD8a (SK1: BUV737, BD Biosciences), streptavidin (BV421, BD Biosciences), anti-CXCR5 (MU5UBEE: Super Bright 600, eBioscience), anti-CCR7 (150503: BV711, BD Biosciences), anti-CD4 (L200: BV786, BD Biosciences), anti-CD95 (DX2: PE, BioLegend), anti-CD28 (CD28.2: PE-DAZZ, BioLegend), anti-PD-1 (J105, PerCP-eFluor710, eBioscience), anti-CCR5 (3A9: APC, BD Biosciences), anti-CD3 (SP34-2, Alexa Fluor700, BD Biosciences), and anti-CD20 (2H7: APC/Fire750, BioLegend). Intracellular staining with anti-Ki67 (B56: FITC, BD Biosciences) was performed for 45 min after lyse/Fix (BD Biosciences) and permeabilizations. Polychromatic (8–14 parameter) flow-cytometric analysis was performed on a LSR II BD instrument as previously described.^51^ List mode multiparameter data files were analyzed using the FlowJo software program (Tree Star, v.9.9.6).

### Analysis of the anti-CAR immune response

Heat-inactivated plasma from the infused animals was tested for anti-CAR antibodies using ITS06 CAR T cells or EGFR T cells that had been isolated using MACS after incubation with a biotinylated anti-EGFR/Erbitux prepared using the EZ-Link Sulfo-NHS-LC-Biotin (Thermo Scientific) following the manufacturer’s instructions. Plasma was incubated with the cells for 30 min at room temperature followed by staining with an Alexa Fluor 647-anti-IgG antibody (clone M1301G05, Biolegend) for 15 min at room temperature before analysis on a LSR II BD flow cytometer. To test the anti-CAR T cell cellular immune response, CD3^+^ T cells were isolated from RM33782 PBMCs collected at the indicated time using immunomagnetic beads (Easy Sep NHP, STEMCELL Technologies). These effector CD3^+^ T cells were mixed with CellTrace Far Red (Thermo Fisher)-labeled CAR T or EGFR T target cells at an E:T ratio of 1:30 in triplicate wells and analyzed as described above in the real-time killing assay immediately after preparation or after a 2-week stimulation with targets. To stimulate possibly scarce anti-CAR T cells, purified CD3^+^ T cells were cocultured with mitomycin-treated autologous CAR T cells for a total of 2 weeks with replenishment of target cells every 4 days and then tested for the killing of fluorescently labeled CAR T cells.
